# BanglaRegionalTextCorpus: A curated dataset for four regional bangla dialects with standard Bangla and English translation

**DOI:** 10.1016/j.dib.2026.112585

**Published:** 2026-02-11

**Authors:** Md. Tofael Ahmed, Zannatul Mawa Koli, Azmain Mahtab Rahat, Taslima Akhter, Umme Ayman

**Affiliations:** a Department of Computer Science and Engineering. Daffodil International University, Bangladesh; b Department of Information and Communication Technology, Comilla University, Bangladesh

**Keywords:** Bangla language, Bangla regional language, Bangla text classification, Text classification, Natural language processing

## Abstract

The BanglaRegionalTextCorpus is introduced as a curated dataset documenting four regional Bangla dialects: Rangpur, Barisal, Narail, and Khulna along with their corresponding Standard Bangla and English translations. The corpus contains 4653 manually validated sentences, collected from community interactions, field recordings, and publicly available digital sources. Rigorous pre-processing steps, including duplicate removal, normalization, and linguistic validation by native speakers, were employed to ensure data accuracy and consistency. This dataset serves as a comprehensive resource for dialect identification, machine translation, and text classification, as well as for research in sociolinguistics and regional language variation. By capturing phonetic, lexical, and syntactic distinctions across four dialects, it enables the development of inclusive and context-aware NLP models for low-resourced languages. Furthermore, the dataset supports comparative linguistic studies between regional and standardized Bangla, contributing to the preservation and computational representation of dialectal diversity. The BanglaRegionalTextCorpus provides a benchmark resource for future research in Bangla NLP, promoting collaboration, cultural preservation, and equitable language technology development across diverse linguistic communities.

Specifications Table

**Table:** 

Subject	Computer Sciences
Specific subject area	Natural Language Processing, Regional Dialect Resources, Bangla Language Technology, Computational Linguistics, Machine Translation, Sociolinguistics
Type of data	Text Files (xlsx-formatted)
Data collection	The dataset was carefully curated to capture the linguistic diversity of Bangla across four regional dialects: Rangpur, Barisal, Narail, and Khulna. Data sources included on-site recordings, community interactions, and publicly available digital content such as social media posts. In total, the dataset contains 4653 sentences, distributed as 1450 (31.2 %) from Rangpur, 1136 (24.7 %) from Barisal, 1150 (24.4 %) from Narail, and 917 (19.7 %) from khulna. Each sentence is paired with its equivalent English translation. To ensure quality and reliability, a multi-step validation process was employed, including duplicate removal, linguistic verification by native speakers, and consistency checks. This dataset serves as a valuable resource for analysing dialectal variation, developing natural language processing applications, and advancing computational research on Bangla.
Data source location	Community interactions and on-site field data from four regions of Bangladesh (Rangpur, Barisal, Narail, Khulna)
Data accessibility	Repository name: Mendeley Data Data identification number: 10.17632/92r62h4k5k.4 Direct URL to data: https://data.mendeley.com/datasets/92r62h4k5k/4

## 1 Value of the Data

• The dataset provides manually validated regional Bangla text from four dialects (Rangpur, Barisal, Narail, Khulna) aligned with Standard Bangla and English, enabling controlled comparison between dialectal and standardized forms.
• Unlike ONUBAD [1], which focuses on dialect-to-standard translation for a limited set of dialects, BanglaDial [2], which is merged and imbalanced, or BdRegionText [3], which is smaller and classification-oriented, this dataset offers balanced sentence-level data, native-speaker validation, and includes underrepresented southern dialects (Narail and Khulna) with consistent multilingual alignment.
• The corpus can be directly used for benchmarking dialect classifiers, including traditional ML and deep learning models, under a reproducible four-class regional classification setting. For example, a researcher can train a four-class dialect identification model using the dataset as a benchmark to compare the performance of traditional machine learning models against transformer-based architectures across regional Bangla varieties.
• The aligned dialect–standard–English structure enables training domain- and region-specific word embeddings and evaluating their impact on downstream tasks such as sentiment analysis, text normalization, and robustness testing across dialects.
• The dataset supports dialect-aware machine translation and normalization, allowing systematic comparison between dialectal input and standardized Bangla output.
• By extending coverage beyond Standard Bangla, the dataset contributes to reducing regional bias in Bangla language technologies, improving fairness and inclusiveness in real-world NLP applications.
• The primary intended users of this dataset include NLP researchers working on low-resource languages, sociolinguists studying regional variation in Bangla, and language technologists developing dialect-aware language models and applications.

## 2 Background

Bangla, spoken by >270 million people worldwide, is a linguistically rich language with diverse regional dialects and expressions. Despite its global significance, Bangla remains underrepresented in natural language processing (NLP), where most benchmark datasets are dominated by English and a few other widely spoken languages. Over the past few years, several efforts have been made to build Bangla corpora for computational linguistics. For instance, ONUBAD introduces a parallel corpus for dialect-to-standard translation covering Chittagong, Sylhet, and Barisal dialects [1], while BanglaDial provides a large-scale merged corpus annotated with multiple Bengali regional dialects to support dialect analysis and identification [2]. Similarly, BdRegionText focuses on regional Bangla text classification using machine learning techniques across selected dialects [3]. Tense-labelled corpora have been introduced to support temporal sentence classification and grammatical analysis [4], while the BanglaBlend dataset provides a large-scale collection of sentences categorized into saint and common forms of Bangla, thereby enabling sociolinguistic and stylistic studies [5]. In the domain of morphological analysis, BanglaLem has addressed the scarcity of lemmatization resources by curating 96,040 inflected words and achieving state-of-the-art results with a transformer-based BanglaT5 model [6].

Although existing datasets provide valuable resources, they largely emphasize specific linguistic phenomena such as tense, formality, lemmatization, or limited dialect translation. None sufficiently capture the dialectal breadth within Bangladesh. In particular, the dialects of Rangpur, Barisal, Narail, and Khulna exhibit distinct phonetic patterns, lexical choices, and syntactic structures that set them apart from Standard Bangla. These regional variations often make communication challenging for speakers outside the respective areas, and NLP models trained exclusively on standardized corpora struggle to generalize effectively. The lack of a curated dataset documenting these four dialects represents a major limitation for developing inclusive and robust Bangla language technologies.

To bridge this gap, the present study introduces a curated regional Bangla dataset that pairs dialectal sentences with their Standard Bangla and English equivalents wherever possible. Unlike existing corpora, it emphasizes dialectal variation as a primary focus. The dataset spans multiple regions such as sentences from Rangpur, Barisal, Narail and Khulna. By offering dialect–standard–English alignments across multiple regions, this dataset provides a valuable benchmark for tasks such as dialect identification, translation, lemmatization, and sociolinguistic analysis. Ultimately, it supports the development of more robust and contextually relevant NLP applications tailored to the linguistic realities of Bangla speakers.

## 3 Data Description

### 3.1 Dataset overview

Each row in the dataset represents a single sentence instance, consisting of a regional Bangla sentence, its Standard Bangla equivalent, an English translation, and an associated regional dialect label. This dataset documents 4653 dialectal Bangla sentences from Rangpur (1450), Barisal (1136), Narail (1150) and Khulna (917), paired with English equivalents. Each sentence underwent pre-processing steps such as duplicate removal, noise filtering, and linguistic validation to ensure consistency and accuracy. The dataset captures natural variations in vocabulary, phonetics, and syntactic structure across these four regions, making it a rich resource for both computational and sociolinguistic studies. Overall, the dataset provides a balanced yet diverse representation of regional Bangla, offering a reliable benchmark for tasks such as dialect identification, translation, and linguistic analysis.

This dataset serves as a crucial resource for advancing research in natural language processing (NLP), dialect studies, and computational linguistics. By documenting variations across regional Bangla, it enables the development of more robust language technologies, including dialect identification, translation, and linguistic analysis. Beyond computational applications, the dataset offers valuable opportunities for interdisciplinary research in fields such as sociolinguistics, education, and psychology, contributing to a deeper understanding of how language diversity shapes communication and identity. The accompanying description outlines the dataset’s structure, statistical properties, and alignment strategy, ensuring that researchers and practitioners can effectively utilize its potential in both academic and applied domains.

### 3.2 File structure and data organization

**Fig. 1:**
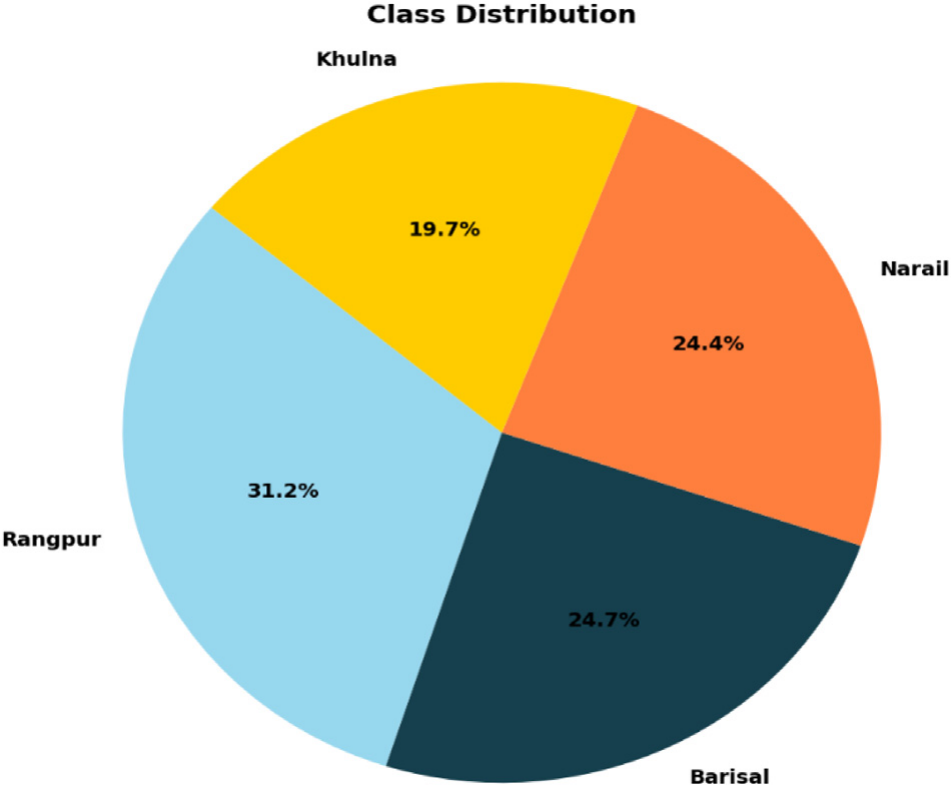
Class distribution of Rangpur, Barisal, Narail and Khulna labels of dataset.

The dataset is distributed in Microsoft Excel (.xlsx) format, where each row corresponds to a single sentence instance. All text fields are encoded in UTF-8 Unicode and normalized using standard Unicode-compliant Bangla script to ensure compatibility across platforms and NLP pipelines. Table 1 summarizes the schema of the BanglaRegionalTextCorpus, outlining the column names, descriptions, and data types. The Regional_Texts column contains original Bangla sentences from four regional dialects, while Standard_Bangla_texts provide their standardized equivalents. The EnglishTranslation column supports cross-lingual analysis. The Region_Labels column assigns each sentence to its corresponding dialect category. This structured schema enables easy reuse and reproducibility for NLP and dialect analysis tasks. Table 2 presents the key components of the dataset, while Fig. 1 illustrates the distribution of sentences across the four regional dialects: Rangpur (31.2 %), Barisal (24.7 %), Narail (24.4 %), and Khulna (19.7 %). This dataset represents a substantial advancement in Bangla regional language resources and establishes a strong foundation for future research in dialectal variation, computational linguistics, and inclusive NLP applications.

**Table 1:** Dataset schema and file format.

Column Name	Description	Data Type
Regional_Texts	Original Bangla regional sentences	texts
Standard_Bangla_texts	Standard Bangla equivalent sentences	texts
EnglishTranslation	English translation	texts
Region_Labels	Regional category	categorical

**Table 2:** Dataset description with attributes and possible values.

*Attribute*	*Description and Possible values*
Regional_Texts	This attribute contains the original Bangla sentences collected from four regional dialects of Bangladesh: Rangpur, Barisal, Narail, and Khulna. Each sentence is paired with its Standard Bangla equivalent and, where applicable, English translation. Example: Rangpur: (Mui boi porber khub bhalobashong) – I love to read books. Barisal: (Tumi aekchher jotnoban manu)-You are a very caring person. Narail: (Se technician asilo)- He was a technician. Khulna: (Tomar dorkar holi eshiane)-If you need anything, come.
Region_Labels	This attribute specifies the dialectal origin of the sentence, with each sentence classified as: Rangpur Barisal Narail Khulna

### 3.3 Sentence length distribution

**Fig. 2:**
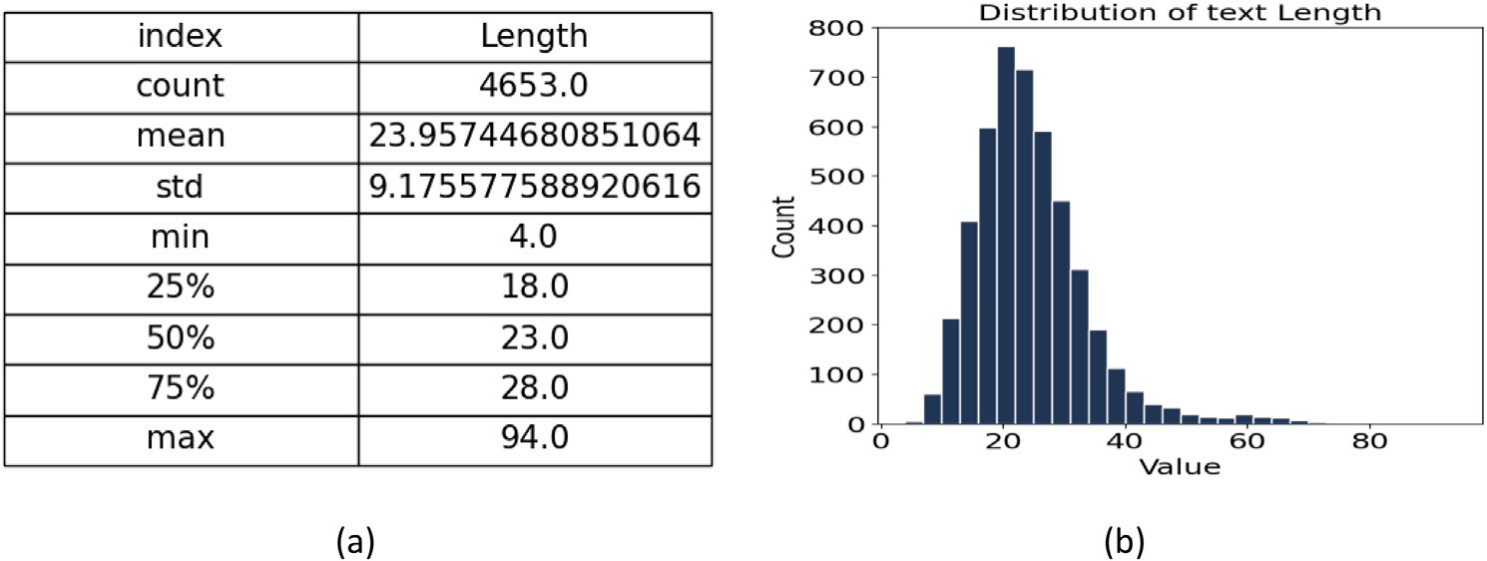
Sentence length analysis of the combined regional dataset (*n* = 4653).

Sentence length is analysed to understand the structural characteristics of regional Bangla expressions in the corpus. Fig. 2 summarizes the overall distribution of sentence lengths across all four regional dialects (Rangpur, Barisal, Narail, and Khulna) which includes 4653 sentences. Sentence lengths range from a minimum of 4 characters to a maximum of 94 characters, with an average of 23.95 characters and a standard deviation of 9.17, indicating moderate variability. The median length is 23 characters, and half of the sentences lie between 18 and 23 characters, showing that most expressions are relatively short and concise. The histogram in Fig. 2 shows a right-skewed distribution, where shorter sentences are far more frequent than longer ones, indicating a predominance of conversational and naturally occurring utterances. The majority of sentences fall within the 15–35-character range, with a sharp peak around 20–25 characters. Sentences exceeding 100 characters are relatively rare, demonstrating that while shorter utterances dominate, a smaller subset of long, detailed sentences contribute to the dataset’s diversity.

Table 3 provides a detailed breakdown of sentence length statistics for the Rangpur, Barisal, Narail, and Khulna dialect subsets. In Rangpur, 1450 sentences are included, with an average length of 23.95 characters and a standard deviation of 9.76, indicating relatively consistent and shorter sentence structures. Most sentences fall between 17 and 28 characters, reflecting concise expressions typical of this region. The Barisal subset, consisting of 1136 sentences, shows similar characteristics with an average length of 23.71 characters (σ = 8.52), where the majority of sentences lie between 18 and 28 characters. The Narail dialect contributes 1150 sentences, slightly longer on average (25.81 characters, σ = 10.20), with most lengths ranging from 19 to 30 characters, indicating moderate variation. In contrast, the Khulna subset displays substantially greater diversity. With 917 sentence Khulna averages 22.81 characters and has a standard deviation (7.10), ranging widely from 6 to 62 characters. This suggests that while other regions favour concise and uniform sentence patterns, Narail dialectal expressions often include longer and more complex structures, contributing to the dataset’s overall richness and variability.

**Table 3:** Statistical analysis of sentence lengths across four regional dialects in the Bangla dataset.

Region	Count	Mean	Std	Min	25 %	50 %	75 %	Max
Rangpur	1450.0	23.39	9.76	5.0	17.0	22.0	28.0	94.0
Barisal	1136.0	23.71	8.52	4.0	18.0	23.0	28.0	70.0
Narail	1150.0	25.82	10.20	7.0	19.0	24.0	30.0	69.0
Khulna	917.0	22.81	7.10	6.0	18.0	22.0	27.0	62.0

**Fig. 3:**
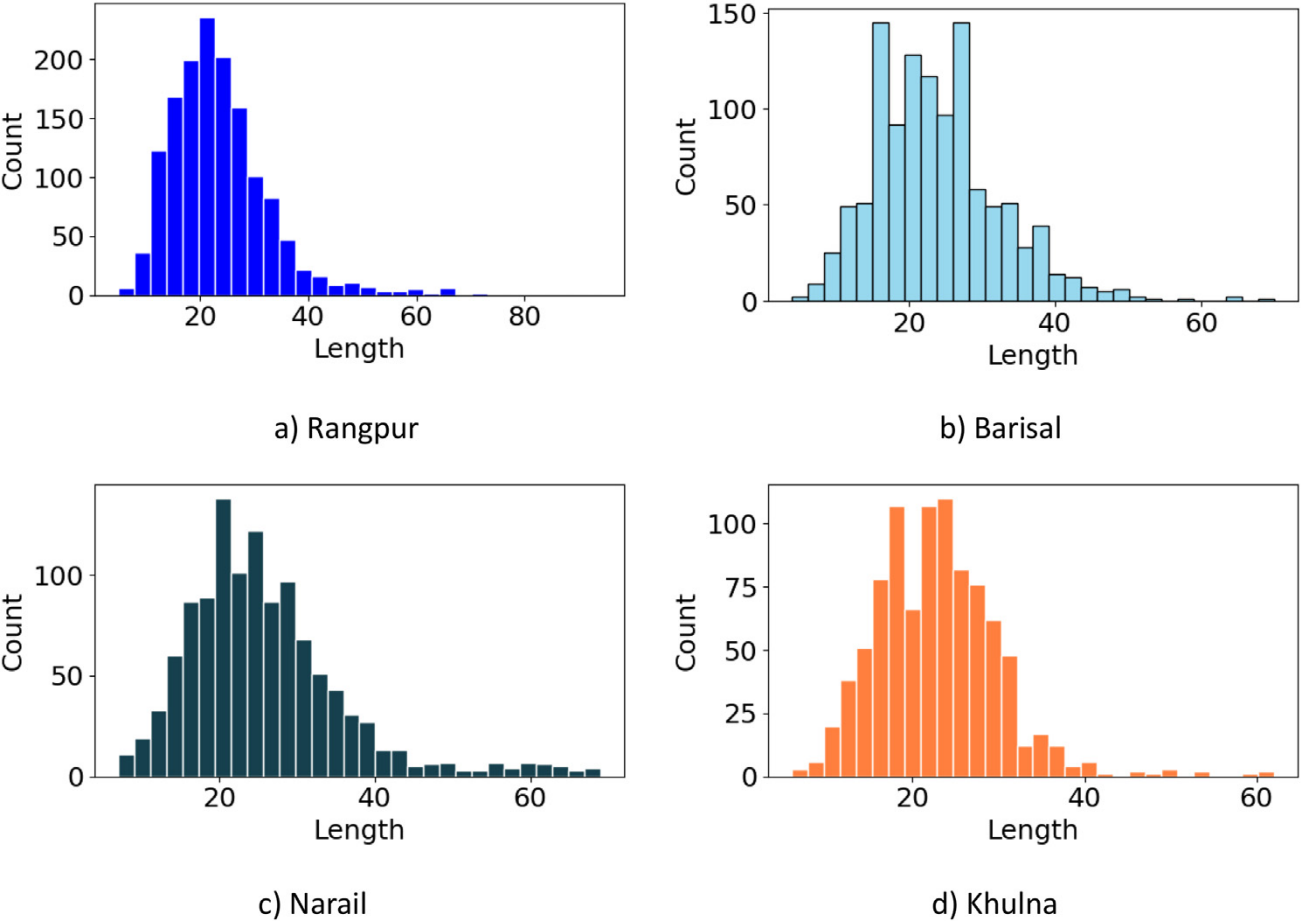
Comparative distribution of sentence lengths across four regional dialects of Rangpur, Barisal, Narail, and Khulna (a-d).

To examine region-specific structural variation, sentence length distributions are compared across the four dialects. The histograms in Fig. 3 highlights notable differences in sentence length distributions across the regions. Rangpur and Barisal exhibit similar patterns, with most sentences clustered between 15 and 30 characters, showing short and relatively uniform structures. Narail also displays a concentration of sentences within the 20–30-character range, though with a slightly longer average length, indicating moderately more variation. In contrast, Khulna demonstrates a markedly different profile: while shorter sentences are present, the distribution is more spread out, extending beyond the average sentence length and reaching up to the maximum observed length in the dataset. This long-tailed pattern reflects the greater syntactic complexity and variability of Khulna dialect sentences compared to the more concise structures observed in Rangpur, Barisal, and Narail.

### 3.4 Lexical statistics and vocabulary analysis

**Fig. 4:**
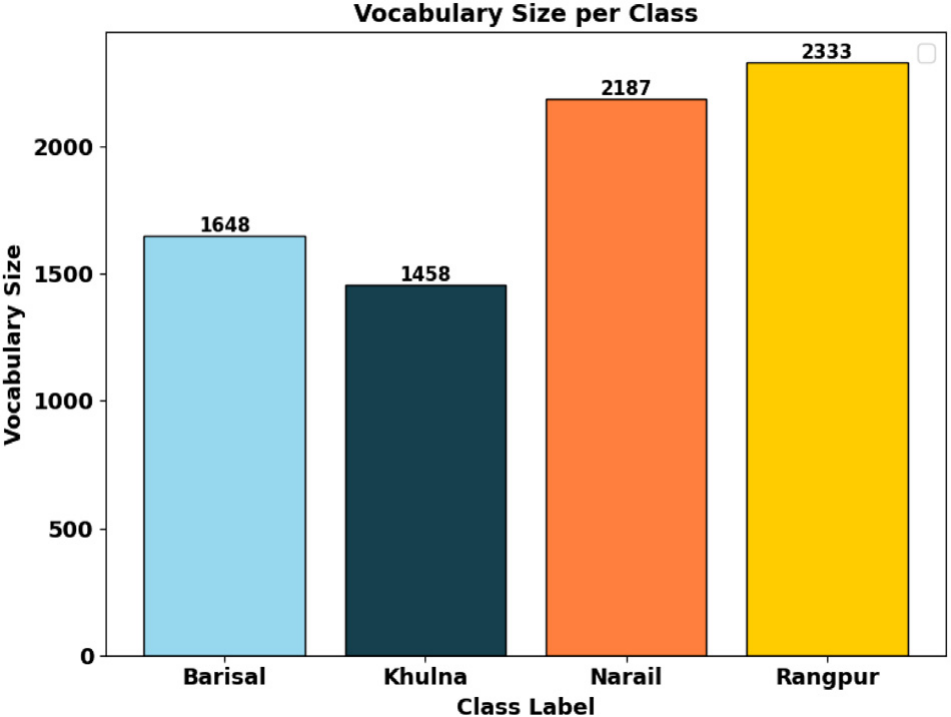
Vocabulary sizes per class.

Lexical diversity across regions is examined by comparing vocabulary sizes for each dialect. The bar chart in Fig. 4 compares the vocabulary size of the dataset across Barisal, Narail, Khulna, and Rangpur. Khulna has the smallest vocabulary with 1458 unique words, followed by Barisal (1648) and Narail (2333). Rangpur exhibits the largest vocabulary size, with 3941 unique words, nearly double that of Khulna. This variation highlights the linguistic diversity of the regions, with Rangpur is contributing more lexical richness and complexity compared to the other dialects.

Table 4 presents the top 20 most frequent words identified across the four dialectal subsets: Rangpur, Barisal, Narail, and Khulna, highlighting distinctive lexical patterns within regional Bangla speech. In Rangpur, personal pronouns such as  (206),  (182), and  (171) appear most frequently, indicating a strong informal and interpersonal tone that characterizes everyday communication. The Barisal subset emphasizes  (161),  (141), and  (131), reflecting a preference for direct conversational forms and second-person address. In contrast, Narail features frequent use of  (177),  (129), and  (57), suggesting a tendency toward more action-oriented and dynamic sentence constructions. Meanwhile, the Khulna subset is dominated by  (114),  (106),  (92), and  (91), which point to stronger expressions of emotion and self-reference. Collectively, these distributions demonstrate both shared lexical elements, such as , and —and region-specific variations, underscoring how dialectal diversity in Bangla manifests through distinct yet overlapping patterns of everyday speech.

**Table 4:** Top 20 frequent words and their frequencies across four regional dialects.

### 3.5 N-gram frequency analysis

The unigram results in Table 5 show that simple, high-frequency words such as , and  dominate, reflecting the central role of inquiry, denial, and personal address in daily communication. The bigrams in Table 6 introduce more structured conversational units like , and  , capturing the interactive and emotionally expressive nature of speech, where questions and polite forms often shape dialogue. The trigrams in Table 7 further expand these expressions into natural, context-rich phrases such as  and , representing curiosity, emotion, and everyday social interaction. Taken together, these frequency analyses illustrate that across dialects, conversational Bangla is shaped by heavy use of pronouns, interrogatives, and negations, with recurring patterns of questioning and evaluation dominating both short and extended expressions. This consistency across n-gram levels underscores the natural, dialogic nature of regional speech while highlighting subtle lexical differences between regions.

**Table 5:** Top 20 unigrams among all sentences with their frequency.

**Table 6:** Top 20 Bigrams among all sentences with their frequency.

**Table 7:** Top 20 Trigrams among all sentences with their frequency.

### 3.6 Qualitative illustration of regional variation

**Fig. 5:**
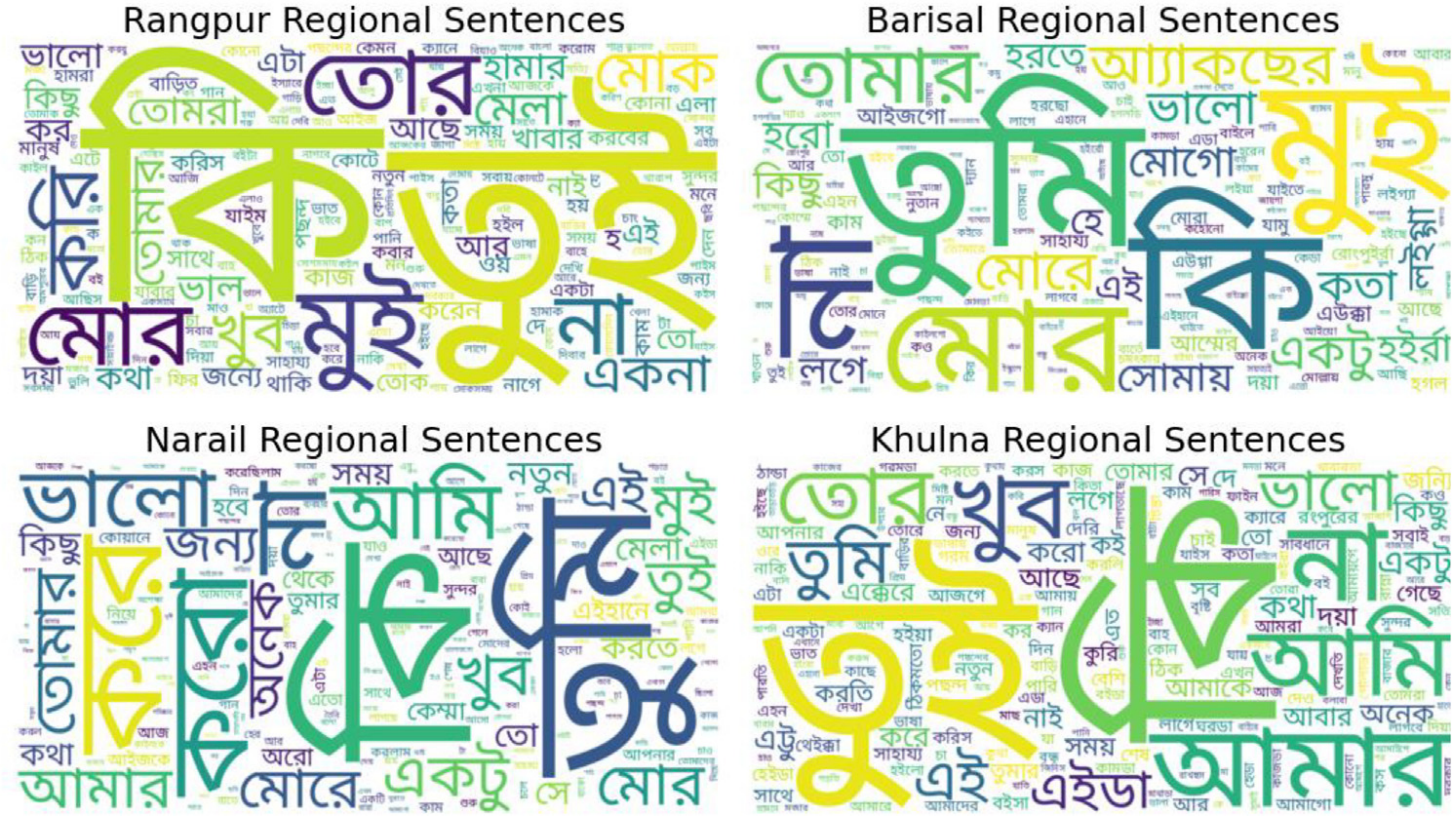
Comparative word cloud representations of frequent words across four Bangla regional dialects (Rangpur, Barisal, Narail, and Khulna).

To provide a qualitative illustration of regional lexical usage, word clouds are generated for each dialect. Fig. 5 presents word clouds for the four regional subsets, Rangpur, Barisal, Narail, and Khulna, showing the most common words people use in everyday conversations. In the Rangpur region, words like  (What),  (You), and (I) stand out, reflecting the friendly and informal way people interact. The Barisal dialect highlights  (You), (I) and  (My), revealing a more direct yet warm conversational style. In Narail, frequent words such as  (What), (I), and  (Good) suggest a curious and expressive tone, while Khulna features  (You),  (What), and  (My), showing both emotional depth and personal connection in speech. Together, these word clouds paint a colourful picture of how people across regions speak naturally, each dialect carrying its own charm, rhythm, and sense of familiarity, yet all sharing the same human warmth of everyday Bangla conversation.

## 4 Experimental Design, Materials and Methods

### 4.1 Data annotation

The annotation of the BanglaRegionalTextCorpus was carried out through a carefully structured manual process to ensure linguistic accuracy and reliability. Sentences were collected from four dialectal regions: Rangpur, Barisal, Narail, and Khulna and each entry was aligned with its equivalent in Standard Bangla and, where possible, English translation. To guarantee consistency, a group of native speakers from each region, along with linguistic experts, participated in the annotation.

During the process, annotators were asked to verify three components: (i) the dialectal sentence, ensuring that it accurately reflected regional speech; (ii) the Standard Bangla equivalent, which preserved meaning while normalizing lexical and syntactic variations; and (iii) the English translation, where applicable, to enable cross-linguistic alignment. Each sentence was reviewed independently by at least three annotators. In cases of disagreement, majority voting was applied, and unresolved conflicts were adjudicated by an expert linguist to minimize subjectivity.

Table 8 demonstrates this annotation workflow, showing how dialectal sentences were validated and mapped to their Standard Bangla and English forms. In most cases, annotators reached strong agreement, underscoring the reliability of the dataset. This systematic approach ensures that the resource captures the richness of regional Bangla while offering standardized representations suitable for a wide range of NLP applications. Table 9 presents the final statistics of the Regional Bangla Dialect Dataset, showing a total of 4653 sentences distributed across four regions: 1450 from Rangpur, 1136 from Barisal, 1150 from Narail, and 917 from Khulna.

**Table 8:** Example of annotation process with majority voting across dialectal sentences.

**Table 9:** Sample entries from the BanglaRegionalTextCorpus.

### 4.2 Data pre-processing

Before annotation and analysis, the raw corpus underwent a rigorous pre-processing pipeline to ensure consistency, clarity, and usability. All pre-processing steps were implemented using customized python scripts provided in Mendeley repository, following these steps: duplicate removal, noise filtering, emoji removal, spelling normalization, and so on. The initial dataset contained dialectal sentences collected from four regions: Rangpur, Barisal, Narail, and Khulna, which varied in spelling, length, and style. To prepare the data, several key steps were applied systematically.

First, duplicates and incomplete entries were removed to avoid redundancy and noise. Sentences containing only symbols, emojis, or non-textual artifacts were excluded to retain meaningful linguistic content. All text was then normalized into Unicode-compliant Bangla script, correcting encoding inconsistencies across sources. Next, punctuation and extraneous markers were removed, except where essential for sentence meaning. Spelling variations, which are common in user-generated and dialectal text, were standardized using a set of manual correction rules and region-specific dictionaries while preserving distinct dialectal forms where necessary. The dataset was further filtered to exclude irrelevant or non-informative items, such as advertisements or unrelated textual fragments. Importantly, stop words were retained to preserve contextual and functional meaning, since their presence in dialectal expressions is crucial for both syntactic and semantic analysis.

Following pre-processing, the corpus was re-validated to ensure that each sentence maintained its dialectal authenticity while being cleaned of noise. The final dataset consisted of 4653 high-quality sentences, aligned with Standard Bangla and, where applicable, English translations, making it suitable for downstream tasks such as dialect identification, translation, and sociolinguistic analysis.

### 4.3 Analysis of proposed dataset

**Fig. 6:**
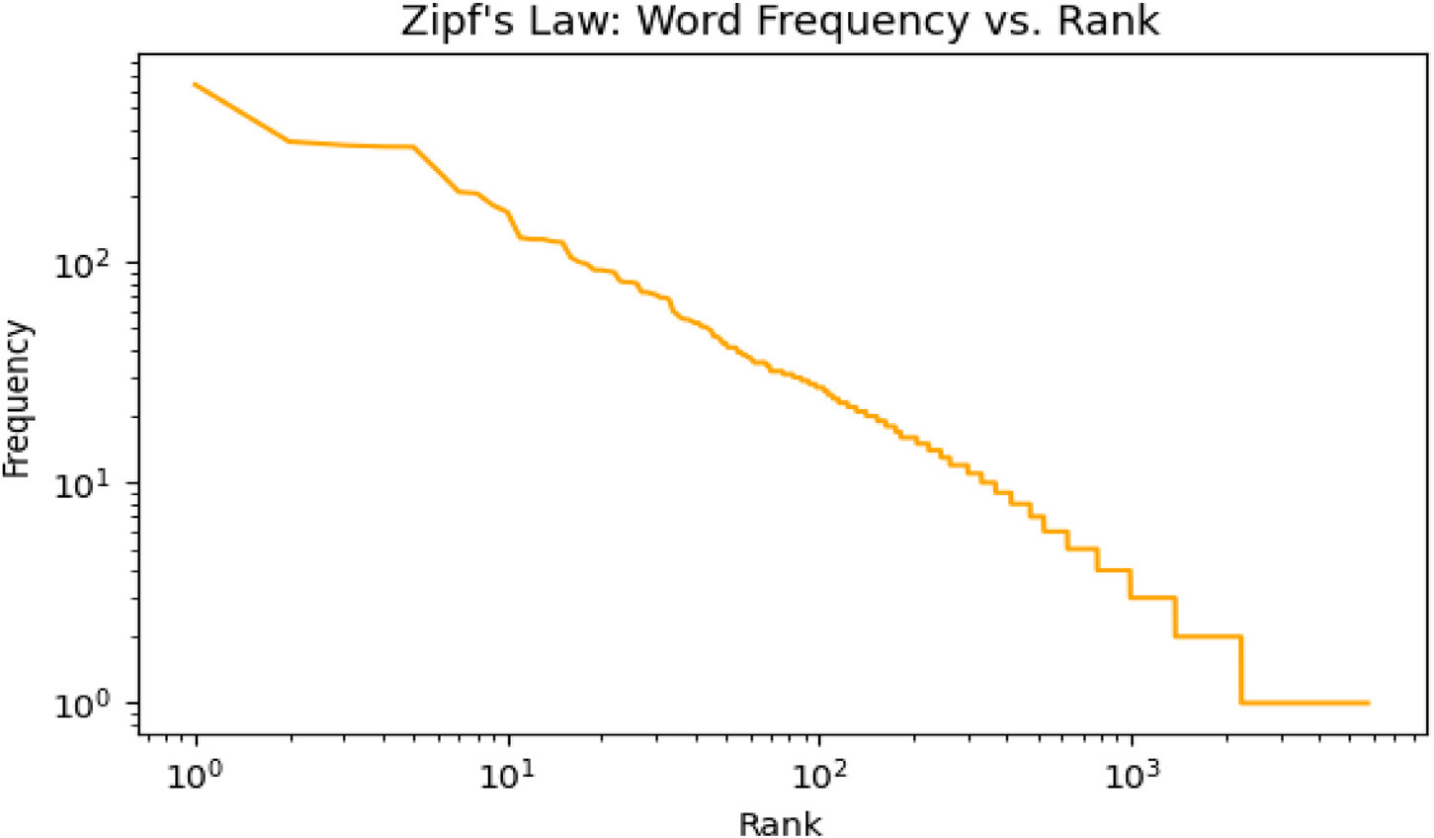
Zipf’s Law applied to the BanglaRegionalTextCorpus.

To analyse the lexical distribution, we applied Zipf’s Law, which follows a power-law relationship indicating that the frequency of a word is inversely proportional to its rank. The plot in Fig. 6 illustrates the relationship between word frequency and rank on a log–log scale, following the characteristic inverse power-law distribution of Zipf’s Law. The curve follows the expected inverse power-law pattern, where a small number of words appear very frequently while most words occur rarely. At the top ranks, highly common words such as  (What),  (No),  (You), and  (You) dominate, reflecting their central role in everyday regional communication. As the rank increases, the curve descends gradually, representing mid-frequency words like (I),  (My), and (very), which maintain conversational variety and expressiveness. Toward the tail, the frequencies drop sharply, capturing rare and region-specific tokens such as  (Very good),  (All), and  (Me), which highlight local lexical distinctiveness across dialects. The smooth decline of the curve and its extended tail confirm that the dataset is both linguistically rich and well-balanced combining frequent functional words with a wide range of low-frequency, dialect-specific vocabulary that authentically represents the lexical behaviour of spoken Bangla.

### 4.4 Dataset validation

The BanglaRegionalTextCorpus was validated by evaluating the performance of three deep learning models: LSTM, Bi-LSTM, and Conv1D on a four-class regional sentence classification task involving Rangpur, Barisal, Narail, and Khulna dialects. Table 10 illustrates the core hyperparameter setting for the models used to validate the curated dataset. The dataset was split into 85 % training and 15 % testing samples using a fixed random seed of 42 to ensure reproducibility and the same seed is used for tokenizer initialization and model training. Word-level tokenization was applied using the Keras Tokenizer with a maximum vocabulary size of 20,000 tokens, and all sequences were post-padded to a fixed length of 50 tokens. A trainable embedding layer with 200 dimensions was used across all models. All models were trained for 50 epochs using the Adam optimizer with a learning rate of 0.001, a batch size of 64, and categorical cross-entropy loss. Ten percent of the training data was reserved for validation, and early stopping was not employed. Performance was evaluated on the held-out test set using accuracy, precision, recall, and F1-score.

**Table 10:** Hyper-parameters and training settings for the models used to validate the Dataset.

Parameter	Value	Parameter	Value
Models	LSTM, Bi-LSTM, and Conv1D	Optimizer	Adam
Train-Test Split	85 % and 15 %	Learning rate	0.001
Random Seed	42	Batch Size	64
Tokenizer	Keras word level	Epoch	50
Vocabulary Size	20,000	Loss function	Categorical cross-entropy
Max. sequence length	50	Early stopping	No
Embedding dimension	200	Validation	10 %

Table 11 provides a detailed comparative evaluation of three deep learning architectures: LSTM, Bi-LSTM, and Conv1D on the *BanglaRegionalTextCorpus* dataset for regional sentence classification. Among the models, the LSTM architecture achieved the highest overall accuracy of 75.79 %, demonstrating notably strong recall for the Rangpur class (0.91) and high precision for Rajshahi (0.90). The Bi-LSTM model followed closely with an accuracy of 74.93 %, exhibiting balanced performance across all classes and maintaining consistent F1-scores for both Rangpur and Rajshahi (0.82 each), indicating its robustness in capturing contextual dependencies in regional text. Meanwhile, the Conv1D model attained a slightly lower accuracy of 73.93 %, performing competitively for Rangpur (Precision: 0.85, F1: 0.83) but showing reduced effectiveness for Narail and Khulna regions, as reflected in their lower F1-scores (0.64 and 0.63, respectively). Overall, the results suggest that while the LSTM model achieved the highest accuracy, the Bi-LSTM model offers more stable and generalizable performance across different regional dialects, underscoring its potential suitability for dialectal text classification in Bangla NLP applications.

**Table 11:** Performance evaluation of deep learning models using BanglaRegionalTextCorpus dataset.

Model	Class	Precision	Recall	F1 Score	Accuracy
LSTM	Rangpur	0.78	0.91	0.84	75.79 %
	Barisal	0.90	0.74	0.81	
	Narail	0.66	0.74	0.70	
	Khulna	0.72	0.60	.65	
Bi-LSTM	Rangpur	0.80	0.84	0.82	74.93 %
	Barisal	0.83	0.81	0.82	
	Narail	0.65	0.68	0.67	
	Khulna	0.70	0.63	0.66	
Conv1D	Rangpur	0.85	0.81	0.83	73.93 %
	Barisal	0.81	0.80	0.81	
	Narail	0.63	0.67	0.64	
	Khulna	0.63	0.64	0.63	

## Limitations

Although the dataset makes a significant contribution to Bangla NLP and dialect research, it has certain limitations. The coverage is restricted to four regions: Rangpur, Barisal, Narail, and Khulna, while other important dialects such as Sylhet and Chittagong are not included. The data is sentence-level only, without extended conversational or discourse contexts, and regional subsets vary slightly in size and lexical richness, with Barisal containing longer and more complex sentences that may affect balance. Some dialectal expressions lack precise Standard Bangla or English equivalents, creating challenges in alignment. Moreover, the dataset excludes sociolinguistic metadata such as speaker demographics and does not provide audio or phonetic information, limiting its scope for speech-related studies. Finally, as it reflects language use from 2024 to 2025, the dataset captures only a temporal snapshot, and may not represent future linguistic changes without further updates.

## Ethics Statement

The regional Bangla dataset was developed in accordance with established ethical standards for linguistic data collection. All sentences were gathered from publicly accessible sources or through direct community interactions with informed consent, ensuring that no personally identifiable information (PII) was included. To safeguard privacy and maintain integrity, the dataset was thoroughly anonymized, and culturally sensitive or inappropriate content was excluded. Annotation and validation were carried out by native speakers following a structured protocol, with consensus-based resolution of disagreements and random quality checks to minimize bias. Ethical considerations guided each stage of data acquisition, preprocessing, and annotation to ensure that the resource remains fair, inclusive, and free from harmful or offensive material.

## Declaration of Generative AI and AI-Assisted Technologies in the Manuscript Preparation Process

During the preparation of this dataset article, the authors used AI-assisted tools such as Grammarly and QuillBot solely to improve the clarity, grammar, and overall readability of the manuscript. These tools were employed for language enhancement purposes only and did not influence the originality, interpretation, or scientific content of the work. After using them, the authors carefully reviewed and revised all sections to ensure that the final version accurately represents their own analysis, findings, and scholarly intent. The authors take full responsibility for the integrity and accuracy of the content presented in this article.

## CRediT authorship contribution statement

**Md. Tofael Ahmed:** Conceptualization, Data curation, Validation, Visualization, Software, Resources, Methodology. **Zannatul Mawa Koli:** Data curation, Validation, Methodology, Writing – original draft, Writing – review & editing. **Azmain Mahtab Rahat:** Validation. **Taslima Akhter:** Data curation. **Umme Ayman:** Supervision, Writing – review & editing.

## Data Availability

Mendeley DataBanglaRegionalTextCorpus: A Curated Dataset for Four Regional Bangla Dialects (Original data).
